# Linkage of Type I Interferon Activity and TNF-Alpha Levels in Serum with Sarcoidosis Manifestations and Ancestry

**DOI:** 10.1371/journal.pone.0029126

**Published:** 2011-12-14

**Authors:** Nadera J. Sweiss, Wei Zhang, Beverly S. Franek, Silvia N. Kariuki, David R. Moller, Karen C. Patterson, Peggy Bennett, Lakshmi R. Girijala, Vaisak Nair, Robert P. Baughman, Joe G. N. Garcia, Timothy B. Niewold

**Affiliations:** 1 Section of Rheumatology, Department of Medicine, University of Illinois at Chicago, Chicago, Illinois, United States of America; 2 Section of Pulmonary, Critical Care and Sleep Medicine, Department of Medicine, University of Illinois at Chicago, Chicago, Illinois, United States of America; 3 Institute for Personalized Respiratory Medicine, University of Illinois at Chicago, Chicago, Illinois, United States of America; 4 Department of Pediatrics, University of Illinois at Chicago, Chicago, Illinois, United States of America; 5 Institute of Human Genetics, University of Illinois at Chicago, Chicago, Illinois, United States of America; 6 Section of Rheumatology, Department of Medicine and Gwen Knapp Center for Lupus Research, University of Chicago, Chicago, Illinois, United States of America; 7 Division of Pulmonary and Critical Care Medicine, Department of Medicine, Johns Hopkins University, Baltimore, Maryland, United States of America; 8 Section of Pulmonary and Critical Care Medicine, Department of Medicine, University of Chicago, Chicago, Illinois, United States of America; 9 Illinois Institute of Technology, Chicago, Illinois, United States of America; 10 Department of Biomedical Engineering, Johns Hopkins University, Baltimore, Maryland, United States of America; 11 Division of Pulmonary and Critical Care, University of Cincinnati, Cincinnati, Ohio, United States of America; University of Pittsburgh, United States of America

## Abstract

**Background:**

Both type I interferon (IFN), also known as IFN-α and tumor necrosis factor alpha (TNF-α) have been implicated in the pathogenesis of sarcoidosis. We investigated serum levels of these cytokines in a large multi-ancestral sarcoidosis population to determine correlations between cytokine levels and disease phenotypes.

**Methods:**

We studied serum samples from 98 patients with sarcoidosis, including 71 patients of African-American ancestry and 27 patients of European-American ancestry. Serum type I IFN was measured using a sensitive reporter cell assay and serum TNF-α was measured using a commercial ELISA kit. Clinical data including presence or absence of neurologic, cardiac, and severe pulmonary manifestations of sarcoidosis were abstracted from medical records. Twenty age-matched non-autoimmune controls were also studied from each ancestral background. Differences in cytokine levels between groups were analyzed with Mann-Whitney U test, and correlations were assessed using Spearman's rho. Multivariate logistic regression models were used to detect associations between cytokines and clinical manifestations.

**Results:**

Significant differences in cytokine levels were observed between African- and European-American patients with sarcoidosis. In African-Americans, serum TNF-α levels were significantly higher relative to matched controls (*P* = 0.039), and patients with neurologic disease had significantly higher TNF-α than patients lacking this manifestation (*P* = 0.022). In European-Americans, serum type I IFN activity was higher in sarcoidosis cases as compared to matched controls, and patients with extra-pulmonary disease represented a high serum IFN subgroup (*P* = 0.0032). None of the associations observed were shared between the two ancestral groups.

**Conclusions:**

Our data indicate that significant associations between serum levels of TNF-α and type I IFN and clinical manifestations exist in a sarcoidosis cohort that differ significantly by self-reported ancestry. In each ancestral background, the cytokine elevated in patients with sarcoidosis was also associated with a particular disease phenotype. These findings may relate to ancestral differences in the molecular pathogenesis of this heterogeneous disease.

## Introduction

Sarcoidosis is a severe multi-system disease of uncertain etiology, thought to be triggered by the aberrant activation of the immune system. The respiratory system is the most frequently involved organ system, with the thorax being afflicted in more than 90% of patients [1]. In extra-pulmonary sarcoidosis, granulomatous inflammation can permeate almost any part of the body, including the skin, liver, joints, muscles, heart, and nervous system [2]. The disease course in sarcoidosis can be acute or chronic, and those subjects with a chronic, progressive course typically carry a worse prognosis with more severe organ system involvement. Those who present with the Lofgren's syndrome (hilar adenopathy, arthritis, and erythema nodosum) typically follow an acute self-limited course; however this subgroup represents a minority of patients with sarcoidosis [1]. It is not yet possible to predict disease course, severity, or the type of organ system involvement for the majority of sarcoidosis patients. Sarcoidosis treatments generally aim to delay or prevent disease progression, which can ultimately lead to organ failure, but as of yet there is not cure for this condition.

Granulomatous inflammation at sites affected by sarcoidosis is marked by the infiltration of activated monocytes, macrophages, and CD4+ helper T cells. Despite this accumulation of immune cells in affected tissues, circulating lymphocyte counts are significantly reduced in many patients [3]. It seems likely that cytokines and chemokines in sarcoidosis not only orchestrate the formation of granulomas locally but also contribute to the egress of immune cells from the peripheral circulation into inflamed tissues. Tumor necrosis factor (TNF)-α and type I interferon (IFN) are cytokines with important roles in coordinating immune reactions, and potentially contribute to the local and systemic inflammatory processes underlying sarcoidosis pathogenesis.

Alveolar macrophages are well recognized as capable of the release of proinflammatory factors including interleukin (IL) 1 and TNF-α that potentially contribute to the underlying disease pathology and inflammation in patients with pulmonary sarcoidosis [4], [5]. TNF-α inhibitors are currently used off-label in sarcoidosis. Small trials and dozens of case studies, including our own, have described mostly positive results [6]–[12]. Infliximab, a chimeric anti-TNF-α monoclonal antibody, was well tolerated and effectively improved index organ involvement without disease recurrence in a case study of refractory sarcoidosis (nine cases of patients who failed to improve despite moderate-to-high prednisone doses) [12]. Long-term follow-up of these patients—as well as some patients treated with the humanized anti-TNF-α monoclonal antibody, adalimumab—revealed that although TNF inhibition is generally safe and effective, it is not curative [13]. In addition, some patients relapsed upon discontinuation of these medications (unpublished data, Sweiss NJ).

Similar to TNF-α, type I IFN has also been implicated in sarcoidosis pathogenesis. Type I IFN plays a critical role in viral defense mechanisms and bridges the innate and adaptive immune defenses [14]. IFN-α contributes to the regulation of immune cells, such as T cells (CD4+, regulatory T cells, and T helper cells), B cells, and dendritic cells [14]. In addition, IFN promotes Th1 immune responses implicated in sarcoidosis pathogenesis. Increased type I IFN signaling has been implicated in a number of autoimmune diseases, including systemic lupus erythematosus [15], Sjogren's syndrome [16], [17], inflammatory myopathies [18], and scleroderma [19]. However, due to its antiviral and immunnomodulatory capacity, IFN-α has also been used to treat chronic hepatitis C (HCV) and recombinant IFN is used as an immunomodulatory treatment for some cancers including melanoma and renal cell carcinoma [20].

Despite the therapeutic utility of IFN-α and TNF-α antagonists in human diseases, these agents have been implicated in the induction of autoimmune diseases [21], including sarcoidosis, with more than 30 cases of TNF-α antagonist-induced sarcoidosis reported [22]–[26]. Similarly, when used to treat HCV, recombinant IFN-α has been associated with sarcoidosis onset [27]. Sarcoidosis has been reported in nearly 100 patients infected with HCV, though not all cases have been directly linked to anti-HCV therapies, and pre-existing sarcoidosis was reactivated in some instances [28]–[33]. A link between these two diseases may exist and warrants further investigation. HCV treatment usually consists of 6–12 months of pegylated IFN-α and ribavirin therapy, which elicit response rates of 40% or more [34], [35]. Together, these two agents increase the Th1-driven immune response, which may account for the onset of sarcoidosis in patients being treated for HCV. Fortunately, most cases of IFN-α-induced sarcoidosis are acute, with cutaneous manifestations that usually disappear spontaneously following the discontinuation of IFN-α and ribavirin.

Physiological crosstalk between TNF-α and IFN-α pathways has been reported. For example in the autoimmune diseases juvenile idiopathic arthritis and Sjogren's syndrome, treatment with anti-TNF-α therapy increased IFN-α signaling in peripheral blood [36], [37]. These findings are supported by *in vitro* studies, which demonstrated that TNF-α regulates IFN-α expression by inhibiting the generation of IFN-α-producing dendritic cells [36].

Given the likely role of TNF-α and IFN-α in sarcoidosis pathogenesis and the urgent need for biomarkers predictive of sarcoidosis phenotype and disease activity, the aim of the present study was to detect associations between serum TNF-α and IFN-α levels and disease phenotypes in different ancestral populations. We hypothesize that such information will shed light on the immune system abnormalities which characterize sarcoidosis, and provide some insights into the pathogenesis of different disease manifestations.

## Materials and Methods

### Patients and Samples

Cytokines were measured in serum samples from 98 patients with sarcoidosis from the Translational Research Initiative in the Department of Medicine (TRIDOM) registry at the University of Chicago. Seventy-one patients were of African-American descent and 27 patients were of European-American ancestry. Clinical data for the sarcoidosis cases were abstracted from medical records including neurologic, cardiac, and severe pulmonary involvement, as well as treatment with TNF-α inhibitors. Neurologic and cardiac involvement was defined as compatible imaging or biopsy studies of the affected organ system in a patient with confirmed sarcoidosis. Severe pulmonary involvement was defined as having a forced vital capacity (FVC) of less than 50% of predicted on pulmonary function testing in a patient with confirmed sarcoidosis. Although patients with <50% FEV1 or DLCO may also be considered severe, we did not take these clinical measures into account for this analysis. We define neurologic, cardiac and severe pulmonary involvement as distinct subphenotypes. In addition, we screened our patients for hepatic involvement and other liver diseases. Among the 98 patients, two individuals were hepatitis C virus positive and three had hepatic sarcoidosis. Clinical and demographic characteristics of the sarcoidosis patient population studied are summarized in Table 1. Age, sex, and self-reported ancestral background were recorded for all subjects in the study. Serum samples were also obtained from a control cohort of 40 age-matched individuals from the TRIDOM registry screened for the absence of autoimmune diseases and the use of corticosteroids for other systemic diseases, as well as significant cardiac, pulmonary or neurologic diseases by medical record review (20 African-American, 20 European-American individuals). The study was approved by the University of Chicago Institutional Review Board, and all subjects provided informed consent.

**Table 1:** Summary of demographic and clinical information for the sarcoidosis patients included in the study.

Characteristics(N = 98)	
**Age, mean (range)**	49.6 (20–83)
**Ancestry and Gender, n (%)**	
European American - Male	9 (9.2)
European American - Female	18 (18.4)
African American – Male	12 (12.2)
African American -Female	59 (60.2)
**Disease Manifestation, n (%)**	
Neurologic	10 (10.2)
Cardiac	11 (11.2)
Pulmonary	84 (85.7)
**FVC <50, n (%)**	18 (18.4)
**Pts on Corticosteroids, n (% of pts with data available)**	
Yes	66 (73.3)
No	22 (24.4)
Past steroid use	2 (2.2)
Unknown, n (% of total pts)	8 (8.1)
**Pts on DMARDs, n (% of pts with data available)**	
Yes	58 (65.2)
Methotrexate, n (% of pts with data available)	26
Others (e.g., azathioprine, leflunomide)	10
Unknown	22
No	31 (34.8)
Unknown, n (% of total pts)	9 (0.9)
**Pts on TNF inhibitors, n (% of pts with data available)**	
Yes	20 (20.4)
No	78 (79.6)

Pts: patients; DMARDs: disease-modifying antirheumatic drugs.

### Reporter cell assay for type I IFN in serum

The assay for type I IFN is fully described and validated in prior reports [15], [38]. Briefly, reporter cells (WISH cells, ATCC #CCL-25) were used to measure the capacity of sera to induce the expression of IFN-regulated genes. After cells were incubated with 50% patient serum for 6 hours, they were lysed, and cDNA was synthesized from total cellular mRNA extracted from the lysates. Real-time PCR was then used to quantify IFN-induced gene expression using the SYBR Green fluorophore system. Primers for the type I IFN-regulated myxovirus resistance 1 (MX1), RNA-dependent protein kinase (PKR), and interferon-induced protein with tetratricopeptide repeats 1 (IFIT1) genes were used, and glyceraldehyde-3-phosphate dehydrogenase (GAPDH) was amplified as a housekeeping gene expression from which to calculate relative expression values. Relative expression levels of these type I IFN-inducible genes were then compared to data from 141 healthy control individuals distinct from the 40 controls tested in this study [15], and the number of standard deviation (SD) above the mean of this control population was used as the type I IFN activity value. For further description of the type I IFN activity score, please see ref. [15]. This assay has been highly informative when applied to multiple different human autoimmune disease populations [15], [17], [18].

### TNF-α ELISA measurement

TNF-α was measured in serum samples using the human monoclonal TNF-α ELISA (Pierce, Rockford, IL), according to the manufacturer's instructions. Our control samples performed similarly to control subject data reported for this ELISA kit in the past (mean of all 40 controls = 1.78 pg/mL, SD = 2.46 pg/mL).

### Statistical analysis

Cytokine data were non-normally distributed, and differences in cytokine levels between groups were analyzed using the non-parametric Mann-Whitney U test. Correlations were assessed using Spearman's rho. For each ancestral group, we used multivariate logistic regression modeling to detect associations between cytokine levels and sarcoidosis organ subphenotypes, and to evaluate for confounding effects of age, sex, and the use of anti-TNF-α therapy. Cytokine data were log transformed prior to be used in the multivariate models. Initial models were tested with one of the three sarcoidosis subphenotypes as the outcome variable, and using age, sex, use of anti-TNF-α therapy, and the other two subphenotypes as predictor variables. Follow-up models discarded predictors with a p value of >0.2, and predictor variables with p<0.05 in follow-up analyses were considered significant. P values shown in the paper are uncorrected for multiple comparisons.

## Results

### Serum cytokine levels in patients with sarcoidosis differ by ancestry

**Figure 1:**
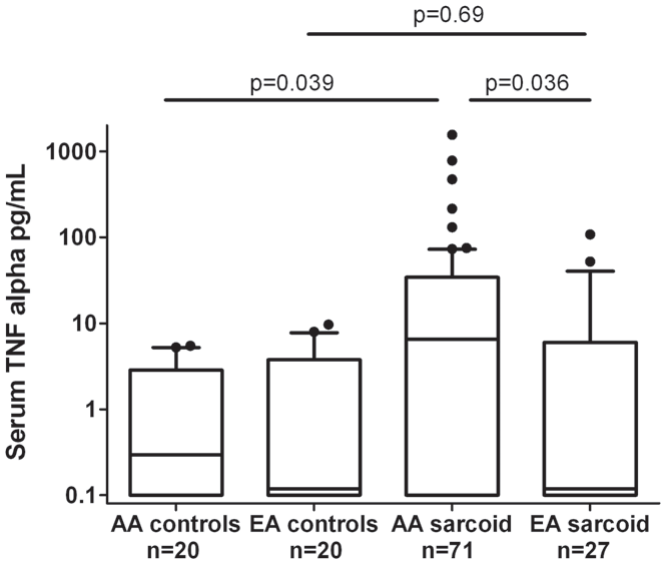
Serum TNF-α levels in sarcoidosis patients and controls in each ancestral background. Lines represent the median, boxes show the interquartile ranges, and error bars show the 10^th^ and 90^th^ percentiles with dots indicating outliers. P-values by Mann-Whitney U test.

**Figure 2:**
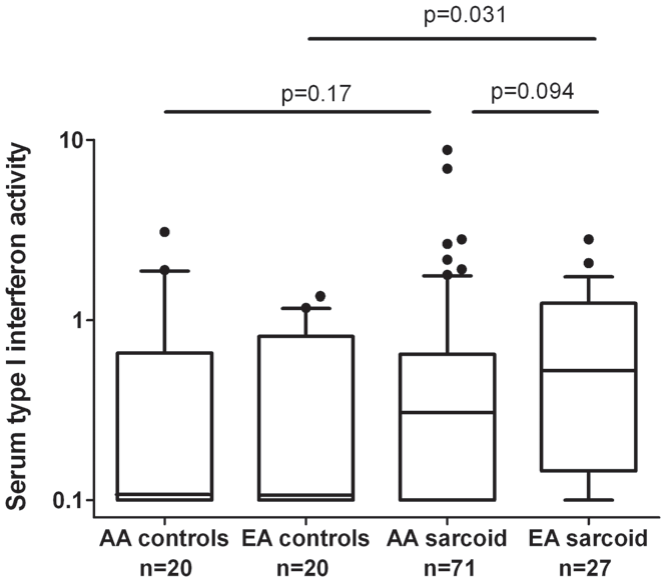
Serum type I IFN activity in sarcoidosis patients and controls in each ancestral background. Lines represent the median, boxes show the interquartile ranges, and error bars show the 10^th^ and 90^th^ percentiles with dots indicating outliers. P-values by Mann-Whitney U test.

No cytokine differences were observed according to gender, but significant differences in TNF-α level were found between sarcoidosis patients of different ancestral backgrounds. As shown in Figure 1, African-American patients had higher TNF-α levels than European-American patients (*P* = 0.036). A non-significant trend was also observed toward higher type I IFN activity in European-American sarcoidosis patients as compared to African-American patients (*P* = 0.094). Controls from each ancestral background were also assessed. African-American sarcoidosis patients had higher TNF-α levels than matched controls (*P* = 0.039, Figure 1), but no significant difference in type I IFN activity was observed between cases and controls. European-American sarcoidosis patients had higher type I IFN activity than did matched controls (*P* = 0.031, Figure 2), but demonstrated no significant difference in TNF-α levels. Notably, no major changes were observed when the subjects on TNF-α inhibitors were removed (Figure S1), suggesting that the correlations we observed between cytokine levels and clinical phenotypes were not due to the treatment with TNF-α inhibitors.

### Multivariate analysis to detect associations between serum cytokine levels and sarcoidosis subphenotypes

We used multivariate logistic regression modeling to detect associations between cytokine levels and presence or absence of neurologic, cardiac, and severe pulmonary involvement. Given the differences observed above in cytokine levels between ancestral backgrounds, regression models were tested in each ancestral background separately. In African-American patients with sarcoidosis, neurologic disease was associated with higher TNF-α (*P* = 0.041) and male sex (*P* = 0.049), and these two associations were independently significant (Table 2). Also in African-American patients, severe pulmonary disease was associated with use of anti-TNF-α agents, and there was a non-significant trend toward association with male sex (*P* = 0.06). In European-Americans, no significant associations were observed in logistic regression models (data not shown), although this may be due to the small number of subjects available in this ancestral background. We did observe non-significant trends toward association of cardiac and neurologic disease with higher serum type I IFN in European-American patients. No significant differences were observed in serum type I IFN and TNF-α levels related to treatment with DMARDs (disease-modifying antirheumatic drugs) or corticosteroids (*P*>0.05 in each ancestral background, data not shown), and incorporating these data in the regression models did not significantly alter the observed cytokine associations.

**Table 2:** P values obtained from stepwise multivariate logistic regression modeling of sarcoidosis phenotypes in African-American sarcoidosis patients.

Predictor variables	Outcome variables in initial model			Outcome variables in follow-up model		
	Neuro	Cardiac	Severe Pulm	Neuro	Cardiac	Severe Pulm
Neuro	X	0.15	>0.2	X	0.29	X
Cardiac	0.13	X	>0.2	0.14	X	X
Severe Pulm	>0.2	>0.2	X	X	X	X
Male Sex	0.062	>0.2	0.06	**0.049**	X	0.06
Age	>0.2	>0.2	>0.2	X	X	X
Anti-TNF-α	>0.2	>0.2	0.02	X	X	**0.026**
IFN	>0.2	>0.2	>0.2	X	X	X
TNF	0.042	0.14	>0.2	**0.041**	0.19	X

Neuro, cardiac, and severe pulm: presence of neurologic, cardiac, and severe pulmonary involvement respectively, as defined in the methods section;

Anti-TNF-α: patient using anti-TNF-α therapy at the time of the study.

IFN and TNF: log transformed values from the serum TNF-α and type I IFN activity assays;

X: not included in the model as a predictor;

numeric values shown indicate the p-value for that variable in the specified logistic regression model.

### Quantitative analysis of cytokine levels in sarcoidosis subphenotypes

**Figure 3:**
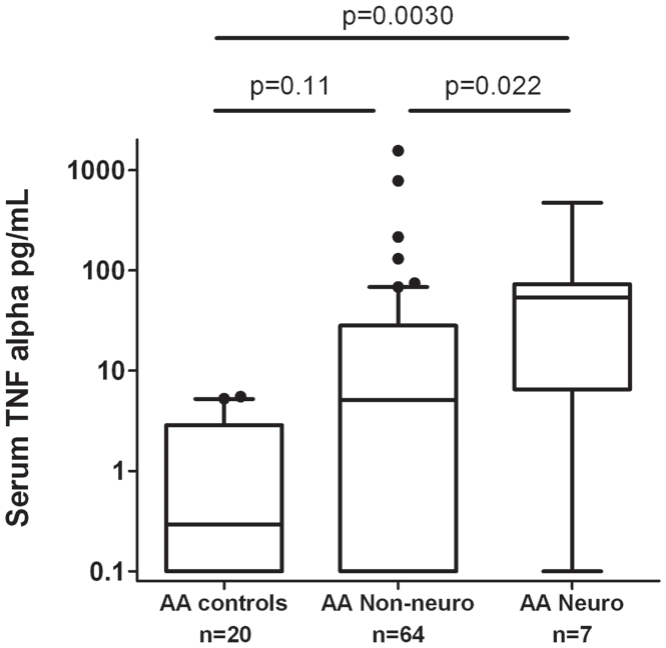
Serum TNF-α levels in African-American controls and sarcoidosis patients stratified by presence or absence of neurologic involvement. Lines represent the median, boxes show the interquartile ranges, and error bars show the 10^th^ and 90^th^ percentiles with dots indicating outliers. P-values by Mann-Whitney U test.

**Figure 4:**
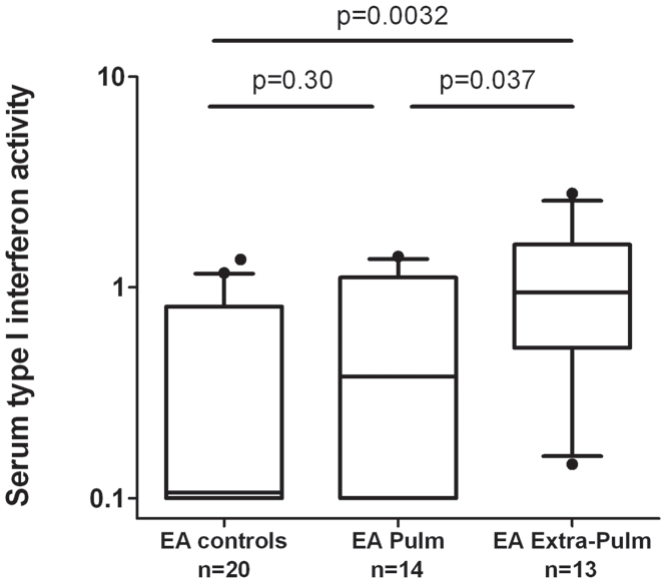
Serum type I IFN activity in European-American controls and sarcoidosis patients stratified by presence or absence of extra-pulmonary manifestations. Lines represent the median, boxes show the interquartile ranges, and error bars show the 10^th^ and 90^th^ percentiles with dots indicating outliers. P-values by Mann-Whitney U test.

Guided by the logistic regressions above, we analyzed quantitative cytokine levels in subgroups suggested by the logistic regression models. In African-Americans, quantitative TNF-α levels were higher in sarcoidosis with neurologic disease compared to patients lacking this manifestation (*P* = 0.022) and to African-American controls (*P* = 0.0030, Figure 3), with the same patterns maintained when the few patients on TNF-α inhibitors were removed (Figure S1). In European-Americans, we found higher type I IFN levels in the group of patients with extra-pulmonary disease compared to patients with lung-limited disease (*P* = 0.037) and to European-American controls (*P* = 0.0032, Figure 4). In addition, the involvement of either central (CNS) or peripheral nervous systems, as well as the occurrences of a depressed ejection fraction (EF) and/or chronic heart failure (CHF) did not appear to affect the observed association trends. Whether these findings can be explained by differences in treatment is unknown and warrants further attention.

## Discussion

To our knowledge, this study is the first to demonstrate ancestral and subphenotype correlations with serum cytokine levels in patients with sarcoidosis. The cytokine which demonstrated significant differences in each ancestral background was also associated with a particular subphenotype. That these associations were distinct by ancestry is intriguing, as there are well-known differences in the prevalence of both sarcoidosis and of particular subphenotypes within sarcoidosis in different ancestral populations [39]. It is possible that the molecular pathogenesis of sarcoidosis also differs significantly by ancestry, and that the cytokine abnormalities we have detected reflected some of the differences in pathogenesis in this heterogeneous disease. If the ancestral differences in sarcoidosis pathogenesis can be understood in greater detail, personalized therapy may become possible, and cytokines and anti-cytokine therapies would likely be important in these treatment decisions.

Although the search for serum biomarkers of neurosarcoidosis had not been previously successful, a recent study found that levels of the soluble IL-2 receptor in cerebrospinal fluid (CSF) may assist in diagnosing patients with or susceptible to neurosarcoidosis [40]. Serum biomarkers of neurologic involvement would also be of great utility. Our data suggest that serum TNF-α may provide some diagnostic information regarding neurologic disease in African-American sarcoidosis patients. Although serum TNF-α would not likely be useful as a single indicator, our results here support its potential use in predictive algorithms. In our own, unpublished clinical experience, neurosarcoidosis is particularly responsive to anti-TNF-α therapy when compared with other sarcoidosis phenotypes. This observation may be related to the elevated serum TNF-α level we found in this sarcoidosis phenotype.

We found high serum type I IFN in a subgroup of patients defined by ancestry and specific disease manifestations. This result is very interesting, as sarcoidosis is reported to result from exogenous administration of type I IFN as a therapy for malignancy or chronic viral infection, and it is possible that higher endogenous level of type I IFN could be related to disease susceptibility as well. This model seems to also apply to systemic lupus erythematosus, as many of the genetic polymorphisms associated with disease susceptibility are also associated with increased serum IFN level [41]–[43].

It is important to note that all patients included in our study were evaluated at a tertiary care center, with the majority treated with corticosteroids (73.1%, Table 1). Two patients had comorbid HCV and two others had a history of prior corticosteroid use. Though other liver diseases have been associated with increased circulating TNF-α level [44], we did not observe unusually elevated TNF-α among our three patients with hepatic sarcoidosis and two patients with positive hepatitis C virus infection. Furthermore, 65.2% of patients were on DMARDs (mostly on methotrexate, with a small proportion of patients on hydroxychloroquine, leflunomide, azathioprine, and cellcept), and 20.4% were receiving TNF inhibitors (Table 1). Notably, the majority the patients treated with DMARDs (82.8%) were on concomitant corticosteroid therapy. We did not observe any overall effects of these treatments on cross-sectional cytokine levels measured in this paper. We cannot rule out the possibility that these treatments could have some impact upon cytokine levels, however these treatments were not responsible for the differences in cytokine levels we observed. Our analyses did not take disease duration into consideration, which may also impact levels of circulating cytokines in patients with sarcoidosis. Whether TNF-α and type I IFN are persistent and stable biomarkers also warrants investigation.

In summary, we identified several significant associations between disease sub-phenotypes and serum levels of TNF-α and type I IFN, which were distinct in different ancestral backgrounds. Particularly, our findings appeared to support that severe sarcoidosis phenotypes have distinct pathogenesis among racial groups, though a larger cohort of patients from different populations may be necessary to confirm this observation. Therapies directed at both of these cytokines are either already in use (TNF-α) or in clinical trials (type I IFN) in autoimmune disease. The results of our study may help guide the potential application of these agents in sarcoidosis, as our results suggest that each of these cytokines may be more important in a particular group of patients with sarcoidosis. Future work, including additional independent cohorts and longitudinal studies, as well as the availability of more comprehensive clinical data (e.g., granuloma burden) will help translate these findings into diagnostic and prognostic algorithms in sarcoidosis.

## Supporting Information

Figure S1 **Association results after removing patients on TNF-α inhibitors.** (A) Serum type I IFN activity is elevated in European-American patients relative to controls; (B) Serum TNF-α level is elevated in African-American patients relative to controls; (C) Serum type I IFN activity is elevated in European-American patients with extra-pulmonary manifestations relative to controls; (D) Serum TNF-α level is elevated in African-American patients with neurologic sarcoidosis relative to controls, as well as in African-American patients with neurologic sarcoidosis relative to patients without neurologic sarcoidosis.
(TIF)

## References

[1] Iannuzzi MC, Rybicki BA, Teirstein AS (2007). Sarcoidosis.. N Engl J Med.

[2] Sweiss NJ, Patterson K, Sawaqed R, Jabbar U, Korsten P (2010). Rheumatologic manifestations of sarcoidosis.. Semin Respir Crit Care Med.

[3] Sweiss NJ, Salloum R, Ghandi S, Alegre ML, Sawaqed R (2010). Significant CD4, CD8, and CD19 lymphopenia in peripheral blood of sarcoidosis patients correlates with severe disease manifestations.. PLoS ONE.

[4] Baughman RP, Strohofer SA, Buchsbaum J, Lower EE (1990). Release of tumor necrosis factor by alveolar macrophages of patients with sarcoidosis.. J Lab Clin Med.

[5] Steffen M, Petersen J, Oldigs M, Karmeier A, Magnussen H (1993). Increased secretion of tumor necrosis factor-alpha, interleukin-1-beta, and interleukin-6 by alveolar macrophages from patients with sarcoidosis.. J Allergy Clin Immunol.

[6] Rossman MD, Newman LS, Baughman RP, Teirstein A, Weinberger SE (2006). A double-blinded, randomized, placebo-controlled trial of infliximab in subjects with active pulmonary sarcoidosis Infliximab therapy in patients with chronic sarcoidosis and pulmonary involvement.. Sarcoidosis Vasc Diffuse Lung Dis.

[7] Moravan M, Segal BM (2009). Treatment of CNS sarcoidosis with infliximab and mycophenolate mofetil.. Neurology.

[8] Garg S, Garg K, Altaf M, Magaldi JA (2008). Refractory vertebral sarcoidosis responding to infliximab.. J Clin Rheumatol.

[9] Baughman RP, Drent M, Kavuru M, Judson MA, Costabel U (2006). Infliximab therapy in patients with chronic sarcoidosis and pulmonary involvement.. Am J Respir Crit Care Med.

[10] Sweiss NJ, Baughman RP (2007). Tumor necrosis factor inhibition in the treatment of refractory sarcoidosis: slaying the dragon?. J Rheumatol.

[11] Sweiss NJ, Curran J, Baughman RP (2007). Sarcoidosis, role of tumor necrosis factor inhibitors and other biologic agents, past, present, and future concepts.. Clin Dermatol.

[12] Sweiss NJ, Welsch MJ, Curran JJ, Ellman MH (2005). Tumor necrosis factor inhibition as a novel treatment for refractory sarcoidosis.. Arthritis Rheum.

[13] Sweiss NJ, Hushaw LL, Curran J, Niewold TB, Baughman RP (2010). TNF inhibition as a novel therapy for refractory sarcoidosis: long term follow up..

[14] Niewold TB, Clark DN, Salloum R, Poole BD (2010). Interferon alpha in systemic lupus erythematosus.. J Biomed Biotechnol.

[15] Niewold TB, Hua J, Lehman TJ, Harley JB, Crow MK (2007). High serum IFN-alpha activity is a heritable risk factor for systemic lupus erythematosus.. Genes Immun.

[16] Bave U, Nordmark G, Lovgren T, Ronnelid J, Cajander S (2005). Activation of the type I interferon system in primary Sjogren's syndrome: a possible etiopathogenic mechanism.. Arthritis Rheum.

[17] Niewold TB, Rivera TL, Buyon JP, Crow MK (2008). Serum type I interferon activity is dependent on maternal diagnosis in anti-SSA/Ro-positive mothers of children with neonatal lupus.. Arthritis Rheum.

[18] Niewold TB, Kariuki SN, Morgan GA, Shrestha S, Pachman LM (2009). Elevated serum interferon-alpha activity in juvenile dermatomyositis: associations with disease activity at diagnosis and after thirty-six months of therapy.. Arthritis Rheum.

[19] Assassi S, Mayes MD, Arnett FC, Gourh P, Agarwal SK Systemic sclerosis and lupus: points in an interferon-mediated continuum.. Arthritis Rheum.

[20] Koon H, Atkins M (2006). Autoimmunity and immunotherapy for cancer.. N Engl J Med.

[21] Niewold TB (2008). Interferon alpha-induced lupus: proof of principle.. J Clin Rheumatol.

[22] Daien CI, Monnier A, Claudepierre P, Constantin A, Eschard JP (2009). Sarcoid-like granulomatosis in patients treated with tumor necrosis factor blockers: 10 cases.. Rheumatology (Oxford).

[23] Massara A, Cavazzini L, La Corte R, Trotta F (2010). Sarcoidosis appearing during anti-tumor necrosis factor alpha therapy: a new “class effect” paradoxical phenomenon. Two case reports and literature review.. Semin Arthritis Rheum.

[24] Dhaille F, Viseux V, Caudron A, Dadban A, Tribout C (2010). Cutaneous sarcoidosis occurring during anti-TNF-alpha treatment: report of two cases.. Dermatology.

[25] O'Shea FD, Marras TK, Inman RD (2006). Pulmonary sarcoidosis developing during infliximab therapy.. Arthritis Rheum.

[26] Josse S, Klemmer N, Moreno-Swirc S, Goeb V, Lequerre T (2009). Infliximab induced skin and pulmonary sarcoidosis in a rheumatoid arthritis patient.. Joint Bone Spine.

[27] Doyle MK, Berggren R, Magnus JH (2006). Interferon-induced sarcoidosis.. J Clin Rheumatol.

[28] Niewold TB, Swedler WI (2005). Systemic lupus erythematosus arising during interferon-alpha therapy for cryoglobulinemic vasculitis associated with hepatitis.. C Clin Rheumatol.

[29] Fantini F, Padalino C, Gualdi G, Monari P, Giannetti A (2009). Cutaneous lesions as initial signs of interferon alpha-induced sarcoidosis: report of three new cases and review of the literature.. Dermatol Ther.

[30] Faurie P, Broussolle C, Zoulim F, Trepo C, Seve P (2010). Sarcoidosis and hepatitis C: clinical description of 11 cases.. Eur J Gastroenterol Hepatol.

[31] Rodriguez-Lojo R, Almagro M, Barja JM, Pineyro F, Perez-Varela L (2010). Subcutaneous Sarcoidosis during Pegylated Interferon Alfa and Ribavirin Treatment for Chronic Hepatitis.. C Dermatol Res Pract.

[32] Shinohara MM, Davis C, Olerud J (2009). Concurrent antiphospholipid syndrome and cutaneous [corrected] sarcoidosis due to interferon alfa and ribavirin treatment for hepatitis C.. J Drugs Dermatol.

[33] Ramos-Casals M, Font J (2005). Extrahepatic manifestations in patients with chronic hepatitis C virus infection.. Curr Opin Rheumatol.

[34] Torriani FJ, Rodriguez-Torres M, Rockstroh JK, Lissen E, Gonzalez-Garcia J (2004). Peginterferon Alfa-2a plus ribavirin for chronic hepatitis C virus infection in HIV-infected patients.. N Engl J Med.

[35] Manns MP, McHutchison JG, Gordon SC, Rustgi VK, Shiffman M (2001). Peginterferon alfa-2b plus ribavirin compared with interferon alfa-2b plus ribavirin for initial treatment of chronic hepatitis C: a randomised trial.. Lancet.

[36] Palucka AK, Blanck JP, Bennett L, Pascual V, Banchereau J (2005). Cross-regulation of TNF and IFN-alpha in autoimmune diseases.. Proc Natl Acad Sci U S A.

[37] Mavragani CP, Niewold TB, Moutsopoulos NM, Pillemer SR, Wahl SM (2007). Augmented interferon-alpha pathway activation in patients with Sjogren's syndrome treated with etanercept.. Arthritis Rheum.

[38] Hua J, Kirou K, Lee C, Crow MK (2006). Functional assay of type I interferon in systemic lupus erythematosus plasma and association with anti-RNA binding protein autoantibodies.. Arthritis Rheum.

[39] Sharma OP (2008). Sarcoidosis around the world.. Clin Chest Med.

[40] Petereit HF, Reske D, Tumani H, Jarius S, Markus Leweke F (2010). Soluble CSF interleukin 2 receptor as indicator of neurosarcoidosis.. J Neurol.

[41] Kariuki SN, Niewold TB (2010). Genetic regulation of serum cytokines in systemic lupus erythematosus.. Transl Res.

[42] Kariuki SN, Franek BS, Kumar AA, Arrington J, Mikolaitis RA (2010). Trait-stratified genome-wide association study identifies novel and diverse genetic associations with serologic and cytokine phenotypes in systemic lupus erythematosus.. Arthritis Res Ther.

[43] Salloum R, Franek BS, Kariuki SN, Rhee L, Mikolaitis RA (2010). Genetic variation at the IRF7/PHRF1 locus is associated with autoantibody profile and serum interferon-alpha activity in lupus patients.. Arthritis Rheum.

[44] Bozkaya H, Bozdayi M, Turkyilmaz R, Sarioglu M, Cetinkaya H (2000). Circulating IL-2, IL-10 and TNF-alpha in chronic hepatitis B: their relations to HBeAg status and the activity of liver disease.. Hepatogastroenterology.

