# Supplementary figures and images for: Linkage of Type I Interferon Activity and TNF-Alpha Levels in Serum with Sarcoidosis Manifestations and Ancestry
Source: PLoS One. 2011 Dec 14;6(12):e29126. doi: 10.1371/journal.pone.0029126 (PMC3237595; doi:10.1371/journal.pone.0029126)

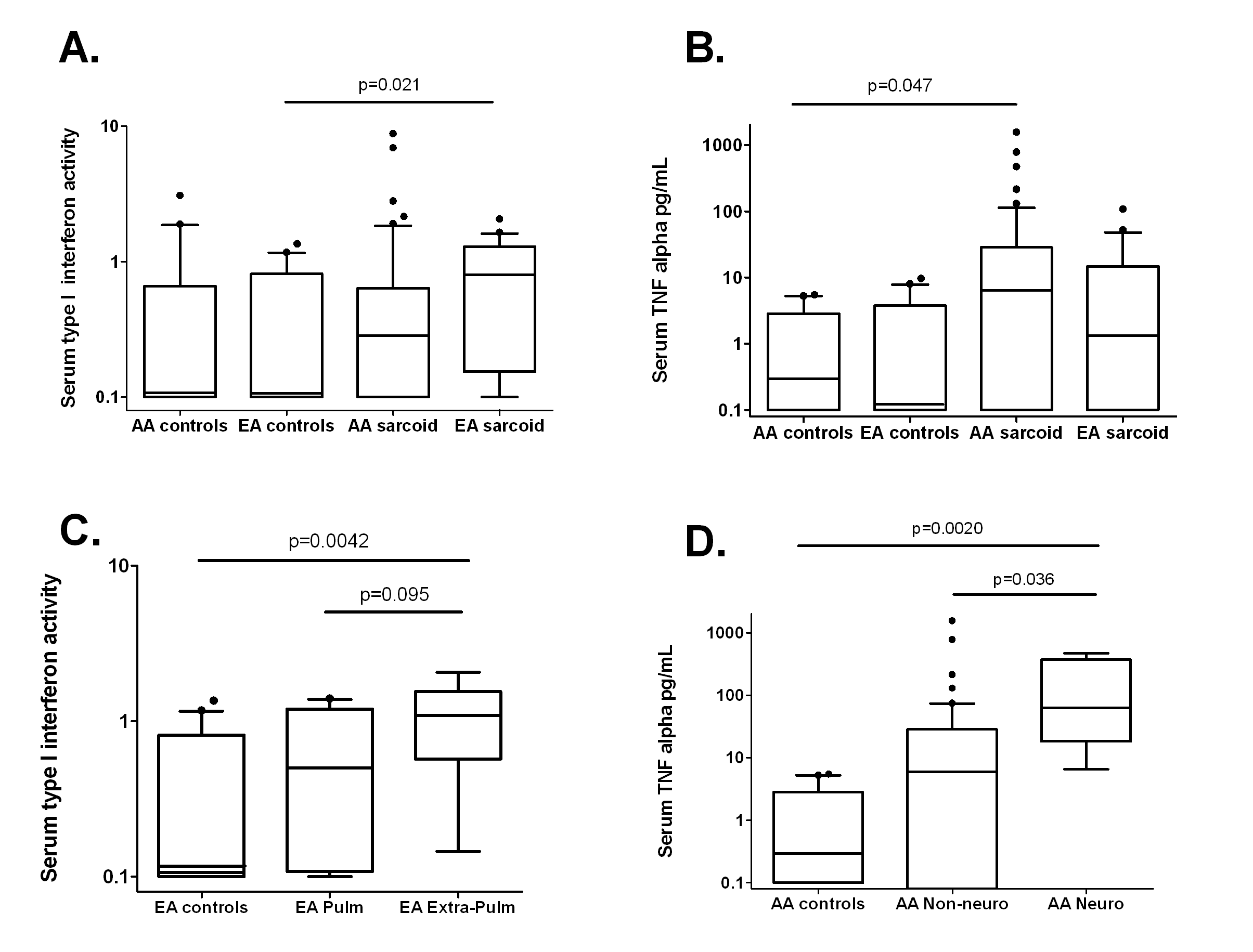

Supplement: Figure S1 — Association results after removing patients on TNF-α inhibitors. (A) Serum type I IFN activity is elevated in European-American patients relative to controls; (B) Serum TNF-α level is elevated in African-American patients relative to controls; (C) Serum type I IFN activity is elevated in European-American patients with extra-pulmonary manifestations relative to controls; (D) Serum TNF-α level is elevated in African-American patients with neurologic sarcoidosis relative to controls, as well as in African-American patients with neurologic sarcoidosis relative to patients without neurologic sarcoidosis. (TIF) [file pone.0029126.s001.tif]
